# Protonic Conduction of Partially-Substituted CsH_2_PO_4_ and the Applicability in Electrochemical Devices

**DOI:** 10.3390/membranes9040049

**Published:** 2019-04-09

**Authors:** Laura Navarrete, Andreu Andrio, Sonia Escolástico, Sergio Moya, Vicente Compañ, José M. Serra

**Affiliations:** 1 Instituto de Tecnología Química, Universitat Politècnica de València—Consejo Superior de Investigaciones Científicas, Av. Naranjos s/n, 46022 Valencia, Spain; launaal@itq.upv.es (L.N.); soesro@itq.upv.es (S.E.); 2 Departamento de Física, Universitat Jaume, I., 12080 Castellón, Spain; aandrio@uji.es; 3 Departamento de Termodinámica Aplicada, Universidad Politécnica de Valencia, C/Camino de Vera s/n, 46022 Valencia, Spain; smoya@ter.upv.es (S.M.); vicomo@ter.upv.es (V.C.)

**Keywords:** cesium dihydrogen phosphate, proton conductor, composite solid electrolyte, conductivity, fuel cell

## Abstract

CsH_2_PO_4_ is a proton conductor pertaining to the acid salts group and shows a phase transition from monoclinic to cubic phase at 232 ± 2 °C under high-steam atmospheres (>30%). This cubic phase gives rise to the so-called superprotonic conductivity. In this work, the influence of the partial substitution of Cs by Ba and Rb, as well as the partial substitution of P by W, Mo, and S in CsH_2_PO_4_ on the phase transition temperature and electrochemical properties is studied. Among the tested materials, the partial substitution by Rb led to the highest conductivity at high temperature. Furthermore, Ba and S-substituted salts exhibited the highest conductivity at low temperatures. CsH_2_PO_4_ was used as electrolyte in a fully-assembled fuel cell demonstrating the applicability of the material at high pressures and the possibility to use other materials (Cu and ZnO) instead of Pt as electrode electrocatalyst. Finally, an electrolyzer cell composed of CsH_2_PO_4_ as electrolyte, Cu and ZnO as cathode and Pt and Ag as anode was evaluated, obtaining a stable production of H_2_ at 250 °C.

## 1. Introduction

Currently, the most-practiced proton conductors used as electrolytes in fuel cells consist of (1) polymers such as Nafion [1], where the operation is limited to low temperatures (~80 to 150 °C), and (2) proton conductors oxides, which operate typically at high temperatures (>500 °C) [2,3,4]. Unlike Proton Exchange Membrane Fuel Cells (PEMFC), based mainly on Nafion and Pt as electrode catalyst, present good performance, the CsH_2_PO_4_-based fuel cells can operate at temperatures above 250 °C, and consequently exhibit higher tolerance to the catalyst poisoning, particularly when CO is used in the fuel due to the higher operation temperature [5,6]. In addition to these properties, higher operation temperature would further improve the catalyst kinetics, allowing the reduction of precious metals load or the utilization of alternative catalysts in the electrodes. In this context, the acid salt CsH_2_PO_4_ (CDP) is a potential candidate as protonic electrolyte for intermediate temperature (around 250 °C) fuel cells [7,8]. CDP exhibits the so-called superionic phase transition [5,9] leading to an abrupt increase in proton conductivity. This jump in protonic conductivity is associated with a polymorphic structural phase transition from a room temperature monoclinic (P21/m) phase to a high-temperature dynamically-disordered cubic (Pm-3m) phase. High steam supply (>30% pH_2_O) is required to ensure high proton conductivity [5,7,10] and to suppress the compound dehydration promoted at high temperatures [11,12]. In order to reduce the superionic phase transition temperature and extend the temperature stability range, cation and anion substitution has been studied [13]. On the other hand, thin CDP (25 m) has been already used as electrolyte in a fuel cell obtaining power densities of 415 mW·cm^−2^ and it was demonstrated that they can operate by using methanol and ethanol as fuels [5,14].

In this work, the effect of different substituents (Ba and Rb in A position and W, Mo, and S in B position) on transport properties (total conductivity and phase transition) of (CsH_2_PO_4_)_0.8_(A*^n^*H_3-*n*_PO_4_)_0.2_ and (CsH_2_PO_4_)_0.8_(HBO_4_)_0.2_ (where *n* corresponds to the valence of the A elements) solid solutions is investigated by means of electrochemical measurements.

In addition, the use of the well-known CDP as fuel cell electrolyte material is studied as a function of temperature, the catalyst used in the electrodes, and the pressure. The higher operation temperatures, compared to the PEMFC operation range, allow the use of different electrode catalysts instead of the expensive Pt. In the present study, Cu and ZnO have been selected as cheaper electrocatalyst alternatives. Several studies using Cu and ZnO as a catalyst for different reactions [15,16] or Cu as electrocatalyst in fuel cell operation mode have outlined the important effect of pressure [15,17], i.e., high pressure promotes the activation of the catalyst leading to faster kinetics and higher cell performance. In order to assess this effect on the designed cells, the system pressure influence has been also evaluated.

Finally, electrolysis was also performed by using CDP as electrolyte. Cu+ZnO has been used as cathode and Pt+Ag as anode.

## 2. Experimental

Different solid solutions based on the partial substitution in A (Ba, Rb) and B (W, Mo, S) positions in (CsH_2_PO_4_)_0.8_(A*^n^*H_3-*n*_PO_4_)_0.2_ and (CsH_2_PO_4_)_0.8_(HBO_4_)_0.2_ systems (where *n* corresponds to the valence of the A elements) were synthesized, resulting in the compounds listed in Table 1.

CsH_2_PO_4_ as powder was prepared from aqueous solutions of Cs_2_CO_3_ and H_3_PO_4_ in a molar ratio of 1:2 [5]. Ba and Rb based compounds were synthesized by using the adequate concentrations of BaCO_3_ and Rb_2_CO_3_, respectively. For the partial substitution in the B-site, H_3_W_12_PO_40_·*x*H_2_O, H_3_Mo_12_PO_40_·*x*H_2_O and H_2_SO_4_ were employed. Precipitation was obtained by adding ethanol. Finally, the obtained powders were dried at 150 °C.

X-ray diffraction (XRD) was used to characterize the phase structure of the samples. XRD measurements were recorded in the 2*θ* range from 20 to 70° on CubiX FAST equipment using CuK*_α_*_1,2_ radiation, and patterns were analyzed using X’Pert Highscore Plus software (PANalytical).

Disk samples used for electrochemical measurements with a diameter of 13 mm and thickness of 1.6 mm were performed by uniaxial pressing. For that purpose, electrodes made of carbon paper were attached to both sides of the electrolyte by co-pressing the synthesized materials and two carbon papers in a sandwich cell configuration.

Electrochemical impedance measurements were performed by using a Novocontrol broadband dielectric spectrometer (Hundsangen, Germany) integrated by a SR 830 lock-in amplifier with an Alpha dielectric interface with 100 mV amplitude and a frequency window 10^−1^ < *f* < 10^7^ Hz. The experiments were carried out isothermally from 180 °C to 280 °C, increasing the temperature gradually in steps of 10 °C and controlled by a nitrogen jet (QUATRO from Novocontrol) with a temperature error of 0.1 °C. Disk samples were sandwiched between two porous stainless steel circular electrodes and set in a glass tube. An atmosphere composed of 30% H_2_O-70% N_2_ was flowed through the tube at atmospheric pressure.

**Figure 1:**
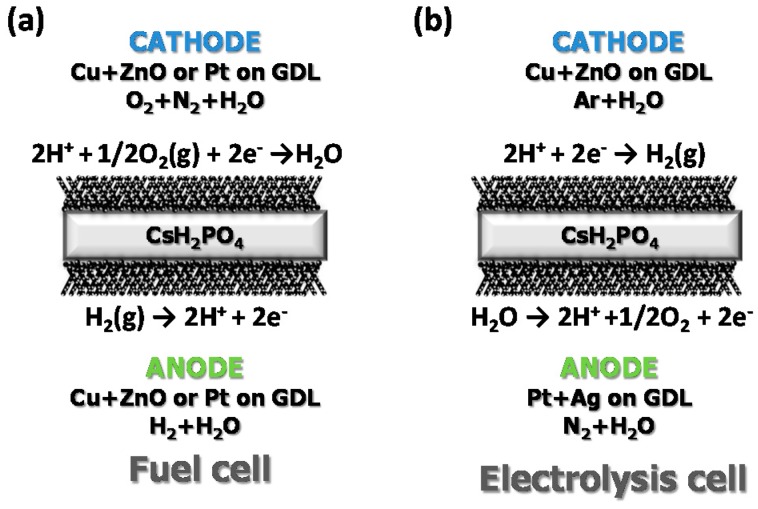
Fuel cell (**a**) and electrolysis cell (**b**) configuration.

After electrochemical characterization of the materials, CsH_2_PO_4_ was tested as electrolyte in a fuel cell and an electrolysis cell under different conditions. For the fuel cell measurement, symmetrical cells (same electrocatalyst for both electrodes) supported on the electrolyte were manufactured. Two different electrodes were prepared: (a) Pt and (b) Cu and ZnO based electrodes. Electrodes were obtained by the infiltration of the abovementioned catalysts on carbon paper (gas diffusion layer-GDL) that was used as backbone. With this purpose in mind, two different solutions in acetone were prepared; hexachloroplatinic acid (0.25 M) for Pt electrode and Cu and Zn precursors (1 M) for Cu and ZnO based electrode. Subsequently, carbon paper was submerged into the solutions and after sonication for 10 min, the samples were dried and treated at 300 °C under reducing atmospheres (pure H_2_) to obtain the different metals (Pt and Cu). Finally, the electrodes and CDP were co-pressed to ensure a good attachment between electrode–electrolyte and a dense electrolyte. Thickness of the electrolytes was 1.8 mm and the active area of the electrodes was 0.64 cm^2^. The fully-assembled fuel cells were measured by means of *i*-*V* curves and electrochemical impedance spectroscopy measurements (EIS). The anode was fed with wet hydrogen (*p*H_2_O ~0.40 bar), whereas wet air (*p*H_2_O ~0.40 bar) was introduced in the cathode. A scheme of the fuel cell configuration is shown in Figure 1a. Experiments were carried out from 250 °C to 280 °C.

For the electrolysis measurements, asymmetric samples were manufactured. Pt solution and Ag paste were used as catalyst in the anode (where wet N_2_ was fed, *p*H_2_O ~0.40 bar), whereas Cu+ZnO were employed in the cathode (where wet Ar is fed, *p*H_2_O ~0.40 bar). After calcination and reduction of both electrodes (GDL+Cu+ZnO and GDL+Pt) at 300 °C in H_2_, infiltrated GDL were pressed with CDP (thickness around 0.9 mm) in a sandwich configuration (see Figure 1b). The current was applied during stages of 30 min and the composition of the cathode outlet gas was monitored by using a mass spectrometer (Pfeiffer Vacuum Omni Star GSD 320 O). Electrolysis measurements were performed at 253 °C and atmospheric pressure. Fuel cell and electrolysis measurements were carried out by a Solartron 1470E/1455 FRA device. Finally, microstructural integrity of the samples was investigated using field emission scanning electron microscopy (FE-SEM) (Zeiss Ultra 55).

## 3. Results

### 3.1. Structural Characterization

**Figure 2:**
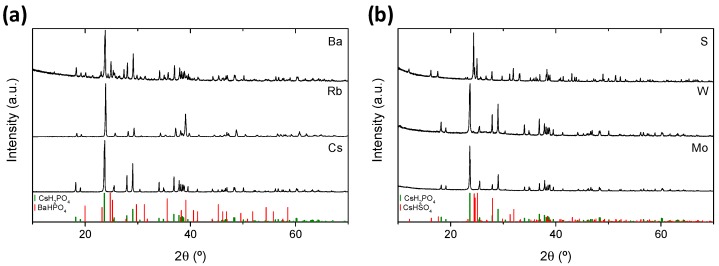
X-ray diffraction (XRD) patterns of (CsH_2_PO_4_)_0.8_(A*^n^*H_3-*n*_PO_4_)_0.2_ (**a**) and (CsH_2_PO_4_)_0.8_(HBO_4_)_0.2_ (**b**) solid solutions.

Room temperature XRD patterns of the A and B partially-substituted solid solutions are shown in Figure 2a,b, respectively. CsH_2_PO_4_ (CDP) is also plotted in Figure 2a for comparison. CDP presents the monoclinic phase (spatial group P21/m) characteristic of the low temperature range. The same occurred for the solid solutions that contain Rb, Mo, and W. The diffraction peaks of the Mo and W substituted compounds shifted to the left indicating higher cell size due to the higher ionic radii of the Mo and W, 0.41 and 0.42 Å (with a coordination number of 4), respectively, as compared with P, 0.17 Å. Conversely, the Rb doped compound presented lower cell size than CDP due to the lower ionic radii of Rb as compared with Cs (1.61 and 1.74 with a coordination number of 8, respectively) [18].

When Cs was partially substituted by Ba, the diffraction peaks corresponding to the monoclinic phase of the CsH_2_PO_4_ and to the orthorhombic BaHPO_4_ phase were detected. Finally, S substituted solid solution presents the CsHSO_4_ monoclinic phase, (space group P21/c), the so-called phase II [19].

### 3.2. Electrochemical Characterization

**Figure 3:**
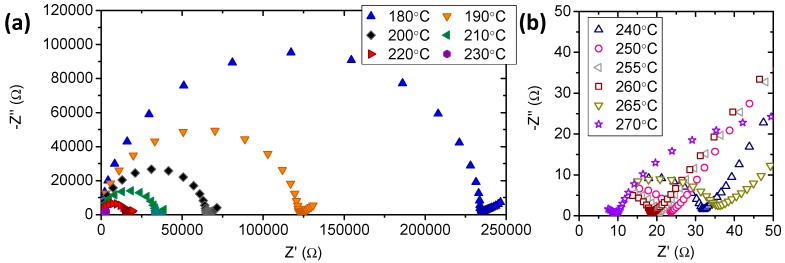
Impedance spectra for CsH_2_PO_4_ compound at the studied temperatures: from 180 to 230 °C (**a**) and from 240 to 270 °C (**b**).

Dielectric impedance spectroscopy measurements were carried out on the different compounds at different temperatures under an atmosphere composed of 30% H_2_O-70% N_2_ aiming to characterize the proton conduction. Impedance spectra for CsH_2_PO_4_ compound at different temperatures calculated from the dielectric permittivity data are shown in Figure 3. The spectra exhibited a large arc at high frequencies (arc in the left side in Figure 3a) that almost extended to the origin. The high frequency intercept of this arc with the real axis can be correlated with the DC conductivity of the different materials. There was a huge decrease in the arc at high frequency with temperature [20]. In fact, a reduction of the resistance around three orders of magnitude was observed above 240 °C. This resistance drop is related with the superprotonic phase transition. Furthermore, the outstanding reduction in the arc at high frequency was accompanied by a reduction of the electrodes resistance (low frequency arc) that became the limiting process above the phase transition temperature.

**Figure 4:**
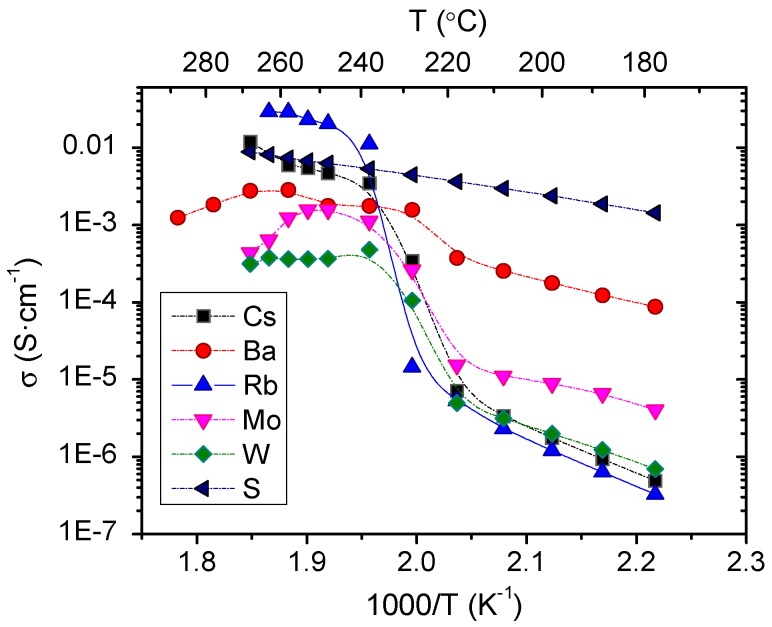
Total conductivity for the different compounds as a function of the temperature.

Conductivity values (S·cm^−1^) were obtained from the fitting of the electrochemical impedance spectra, specifically from the arc that appears at high frequency (with the equivalent circuit RQ, where R indicates resistance and Q capacitance) and corrected by the thickness and the area of each disk sample. The obtained conductivity values, as a function of the temperature, are shown in Figure 4. As a general overview, an important increase in the total conductivity of the studied compounds was observed above 230 °C. As previously mentioned for CsH_2_PO_4_ compound, this improvement in the total conductivity can be ascribed to the transition phase that produces a dramatic increase in the proton conductivity by several orders of magnitude [9]. Depending on the dopant, this increase can occur at a different temperature and the magnitude changes, being very small when S is used as dopant.

An increase of four orders of magnitude in the conductivity of polycrystalline CsH_2_PO_4_ was observed around 240 °C, from 7 × 10^−6^ S·cm^−1^ to 0.03 S·cm^−1^ in agreement with the results observed by Otomo et al. [7]. Concerning Rb based compound, there was an improvement in the conductivity of more than four orders of magnitude around 240 °C. Ikeda et al. [21] tested the influence of Rb contents, showing good conductivity results. Furthermore, they found that the compound with 19% of Rb content presents the highest conductivity in the Rb series (Cs_1-*x*_Rb*_x_*H_2_PO_4_) and the reported conductivity values fit with those obtained in the present work. At temperatures below phase transition, no improvement of the total conductivity was obtained by partial substitution of Cs by Rb. Ba substituted compound presented a conductivity two orders of magnitude higher than the corresponding to the CsH_2_PO_4_ compound at low temperature, whereas above the phase transition temperature, it increased less than one order of magnitude. This behavior can be related with the BaHPO_4_ phase detected by XRD in the compound. BaHPO_4_ presents linear conductivity behavior (almost constant) from 150 to 300 °C [22], thus conferring almost constant conductivity for the Ba-based compound.

On the other hand, when P was partially substituted by Mo and W, the same conductivity behavior, as compared with the parent compound, was observed. The conductivity increased two orders of magnitude in both compounds when the phase transition occurs. Mo doped compound presented higher conductivity below the phase transition temperature as compared with CsH_2_PO_4_, whereas it was lower at high temperature. Furthermore, a decrease in the conductivity was observed above 250 °C.

Finally, S-substituted compound presented different conductivity behavior than the other salts, i.e., no typical transition in the conductivity (steep increase) was observed. This fact is in agreement with the study reported by Ponomareva et al. [13] where the superionic phase transition shifts to lower temperatures and essentially disappears at *x* = 0.15 in the solid solution (CsH_2_PO_4_)_1-*x*_(CsHSO_4_)*_x_*, with the subsequent increase of the conductivity at low temperature.

Apparent activation energies values obtained at low and high temperatures (where low and high temperature means *T* < 230 °C and *T* > 230 °C, respectively) are listed in Table 2. The difference between the activation energy at low and high temperatures can be read in terms of the phase transition. At low temperatures, all samples have higher activation energy ascribed to the monoclinic phase, except for the S-doped compound that possesses a constant activation energy over the studied range of temperatures. When the temperature increased above the phase transition temperature, there was an important reduction in the activation energy that can be attributed to the proton conductivity mechanism in the cubic phase. Under room temperature, there was a network where $PO_{4}^{-3}$ oxyanions were linked via O‒H···O bonds forming H_2_(PO_4_)^−^ layers, and Cs was situated between these layers. When the temperature was increased there was a reorientation of the oxyanions giving rise to transition to the cubic phase. The proton conductivity of CDP at high temperatures (superconductor phase) took place following two different competing mechanisms: Diffusion (vehicle transport) and Grotthus mechanism. In the first one, the fast proton hopping was followed by a slow repositioning of the oxyanions, whereas the reorientation of the PO_4_ group was not necessary in the Grotthus mechanism [23]. At high temperatures, the PO_4_ tetrahedron reorientation (the slowest process) was favored, resulting in the reduction of the energy barrier and the subsequent improvement of the proton transport.

**Figure 5:**
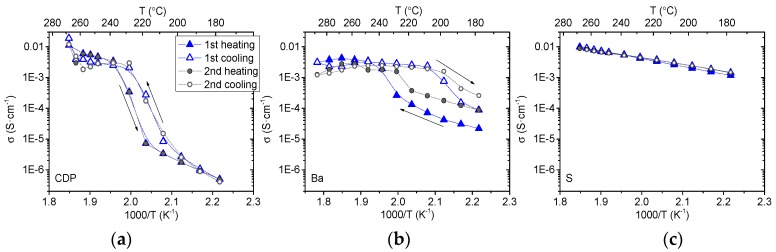
Conductivity of CDP (**a**), Ba (**b**) and S (**c**) substituted compounds as a function of temperature for two heating and cooling cycles.

Conductivity of CsH_2_PO_4_ as a function of the temperature is plotted in Figure 5a for two heating and cooling cycles. The hysteresis observed in the transition behavior, i.e., the difference between the conductivity of CsH_2_PO_4_ measured in the heating and the cooling cycles, can be ascribed to residual surface water present in the grain boundary regions of the polycrystalline material. It has been reported that removal of the residual water requires several days of exposure to temperatures of 205 °C or higher [24]. The results shown in Figure 5 demonstrate that the phase transition from low to high conductive phase took place reversibly, in agreement with the results obtained by Otomo et al. [25]. The same study was performed for Ba and S substituted compounds. Ba exhibited significant hysteresis, whereas it was not found in the S doped compound in the studied range of temperature.

### 3.3. Fuel Cell Measurements

This family of materials exhibits good proton conductivity at high temperatures (>230 °C), and this paves the way for electrochemical cells at medium temperatures. Those operation temperatures can avoid Pt poisoning effect by CO observed in PEMFC. Therefore, the applicability of those materials as electrolyte was assessed in different electrochemical cell configurations.

Although this kind of material has been previously used as electrolyte for fuel cells [5,6,26,27], the present work aims to demonstrate its applicability as electrolyzer for the first time. In order to study this in depth, CsH_2_PO_4_, a material well studied in the literature was selected as electrolyte in different electrochemical applications.

Furthermore, the influence of inexpensive electrocatalysts and higher pressures on the CsH_2_PO_4_-based fuel cells performance was evaluated. For the first objective, Cu and ZnO were selected as alternative catalysts in the electrode because they are cheaper than Pt and would allow for a reduction in the cost of the cell manufacture. In addition, a fuel cell containing Pt in the electrode was also measured for comparison. Moreover, high pressures would allow for the application of them in existing industrial processes, without any intermediate stage.

**Figure 6:**
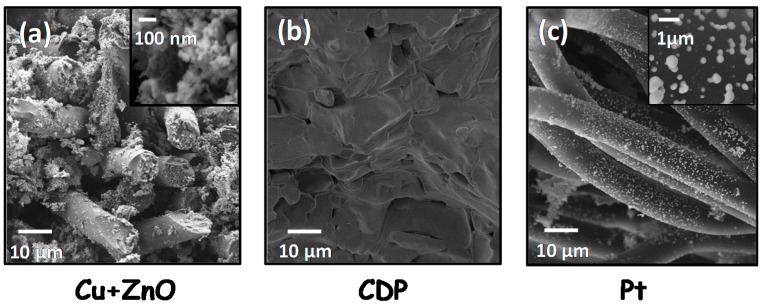
FE-SEM micrographs of the Cu+ZnO based electrode (**a**), dense CDP electrolyte (**b**) and Pt based electrode (**c**).

CsH_2_PO_4_ was tested as electrolyte in a fully-assembled fuel cell. The measured cells consisted of symmetrical cells supported on the electrolyte, i.e., same electrode material was used for both, anode and cathode. Two different cells were manufactured using different electrocatalysts: (a) Cu and ZnO and (b) Pt. SEM micrographs of the Cu+ZnO and Pt-based electrodes supported on carbon fibers (GDL) (diameter ≈ 10 μm) are presented in Figure 6a,c, respectively. Cu+ZnO and Pt particles present nanometric size, but Pt particles are larger (≈150 nm). Furthermore, SEM image of the dense electrolyte is shown in Figure 6b.

**Figure 7:**
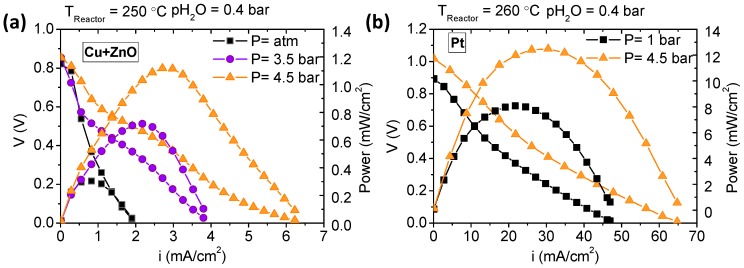
*i*-*V* curves of the Cu-ZnO on GDL/CsH_2_PO_4_/Cu-ZnO on GDL cell at 250 °C (**a**) and Pt on GDL/CsH_2_PO_4_/Pt on GDL cell at 260 °C (**b**) in a fuel cell mode for different total system pressure. Gases: Wet air (cathode) and wet H_2_ (anode).

The electrochemical testing was conducted from 250 °C to 280 °C at different total pressures. 50 mL·min^−1^ of wet hydrogen and wet air (*p*H_2_O ~0.40 bar) were fed in the anode and the cathode chamber, respectively. The performance of the fuel cell with Cu+ZnO based electrodes at 250 °C as a function of the total pressure in the system is shown in Figure 7a. As can be inferred from the graphs, the total pressure played an important role on the fuel cell performance. For instance, the power density of Cu+ZnO based cell increased four times when total pressure was increased from 1 to 4.5 bar. This improvement can be attributed to the higher activity of the Cu+ZnO electrocatalyst under higher pressures. These results are in agreement with the higher activity observed for Cu and ZnO catalysts with the pressure increase improving the reaction rate of several heterogeneous reactions [15,16]. In addition, the preliminary results of Hallinder et al., at different pressures, [17] showed the high effect of pressure on the cell performance. Pressure influence was also studied for the electrode infiltrated with Pt (*T* = 260 °C), and the relative cell performance improvement was smaller than the observed for Cu+ZnO electrode. Indeed, the highest power density was obtained with the Pt catalyst (Figure 7b).

**Figure 8:**
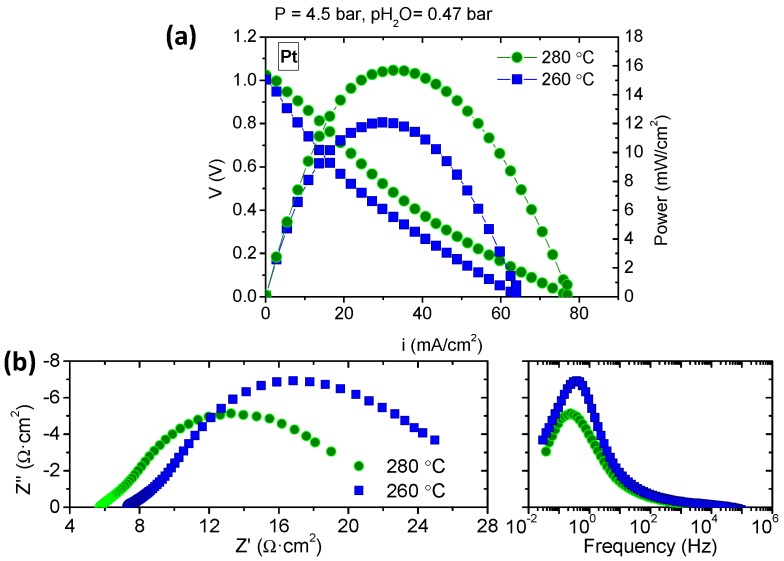
*i*-*V* curves (**a**) and Nyquist and Bode plots (**b**) in a fuel cell mode of the Pt on GDL/CsH_2_PO_4_/Pt on GDL at 260 and 280 °C.

In addition, the temperature influence on the cell performance was checked for the sample infiltrated with Pt at 4.5 bar and two different temperatures: 260 °C and 280 °C. As can be observed from Figure 8a, the electrochemical performance of the fuel cell was thermally activated, and achieved a higher power density at 280 °C. In order to check the nature of the rate limiting steps in the fuel cell performance, electrochemical impedance spectroscopy (EIS) measurements were carried out near the OCV at both temperatures.

The real (ohmic) resistance obtained from the Nyquist plot (Figure 8b) can be assigned to the electrolyte: 7.3 and 5.7 Ω·cm^2^ at 260 and 280 °C, respectively. This high resistance can be ascribed to the important thickness of the electrolyte (1.8 mm), since cell was electrolyte-supported. Furthermore, the EIS spectra highlight the high influence of both electrodes (anode and cathode) on cell performance, i.e., the electrodes polarization resistance (~27.5 and ~23.5 Ω·cm^2^, respectively) was four times bigger than the electrolyte resistance (Figure 8b). These results reveal that the fuel cell performance is limited by the electrodes [28] (anode and cathode). This fact can also be seen in the low Pt nanoparticles dispersion, as evidenced by the SEM image (Figure 6c). This resulted in a limited active area for the hydrogen oxidation/reduction, and subsequently in a low performance of the electrodes. Lower particle size and better distribution would enlarge the triple phase boundary (TPB) length along the electrodes and, consequently, would improve their catalytic activity [29]. On the other hand, higher total pressures at constant *p*H_2_O do not affect the proton conductivity of the CDP electrolyte.

### 3.4. Electrolysis Measurements

CsH_2_PO_4_ material could find a good application as electrolyte in an electrolysis cell. In this case, Cu+ZnO electrode was used as cathode and Pt+Ag as anode. Ag was employed in the anode to increase the TPB length of the electrode. Wet N_2_ (*p*H_2_O ~0.40 bar) was fed in the anode, whereas wet Ar (*p*H_2_O ~0.40 bar) was employed in the cathode. Both electrodes were humidified in order to prevent the dehydration of the electrolyte [30].

**Figure 9:**
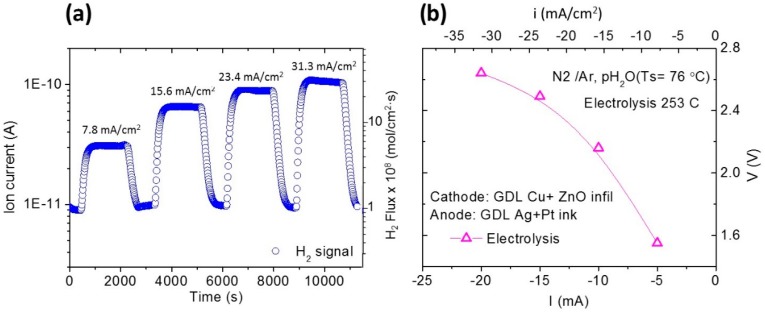
H_2_ signal of the cathode outlet as a function of the applied current (**a**) and *i*-*V* curve of the electrolysis cell (**b**) at 253 °C and atmospheric pressure.

The electrolysis cell was tested at 253 °C and atmospheric pressure and the current was applied during stages of 30 min. H_2_ production was stable when the current was applied to the cell as can be observed in Figure 9a. Nevertheless, a high overpotential can be inferred from the *i*-*V* curves (Figure 9b), which limited the range of the current densities applicable in electrolysis operation. Note that the equilibrium voltage (OCV) for these operation conditions was around 1.075 V.

Due to the good proton conductivity ascribed to CsH_2_PO_4_, the high overpotential can be mainly related to the electrode polarization resistance. The modest electrocatalytic activity and/or bad microstructure of the electrodes can be pointed out as the main reasons. However, it should be noted that these results demonstrate the applicability of CsH_2_PO_4_ for electrolysis around 250 °C, a temperature range with high potential for integration in different industrial process, e.g., direct CO_2_ to hydrocarbons (CH_4_, olefins and alcohols) [31,32].

## 4. Conclusions

The effect of different substituents (Ba and Rb in A position and W, Mo, and S in B position) on transport properties of (CsH_2_PO_4_)_0.8_(A*^n^*H_3-*n*_PO_4_)_0.2_ and (CsH_2_PO_4_)_0.8_(HBO_4_)_0.2_ (where *n* corresponds to the valence of the A elements) solid solutions was investigated by means of electrochemical measurements.

An important increase in the total conductivity of the studied compounds was observed above 230 °C due to the phase transition. This conductivity increase occurred at different temperatures and the magnitude changed depending on the substituent, being very small when S was used as dopant. Furthermore, the reversibility of the phase change of CDP, Ba, and S substituted compounds at low and high temperatures was confirmed.

The performance of a fuel cell with an electrolyte made of CsH_2_PO_4_ was studied under different conditions: temperature, pressure, and employed electrodes (Pt and Cu+ZnO). The high operation temperatures, compared to the PEMFC operation temperatures, allowed the use of cheaper electrode catalysts instead of the expensive Pt. The pressure in the system played an important role in the cell performance, mainly when Cu+ZnO were used as electrodes, reaching a power density four times higher when the system pressure was increased from 1 to 4.5 bar.

Finally, electrolysis was also performed by using CDP as electrolyte. Cu+ZnO was used as cathode and Pt+Ag as anode. Despite high overpotential values being observed, a stable production of H_2_ was obtained, demonstrating the viability of the CDP application in electrolysis cells. Current developments are focused on producing asymmetric cells based on (Rb and S) doped-CDP together with electrodes with improved electrocatalyst activity and mixed protonic-electronic conductivity to minimize polarization resistance during pressurized electrolysis operation. The target electrolysis operation temperature range (220 to 260 °C) will enable a higher degree of thermal integration with catalytic processes such as CO_2_ conversion to hydrocarbons or hydrogenation reactions.

## Figures and Tables

**Table 1:** Nomenclature for the compounds based on the solid solutions (CsH_2_PO_4_)_0.8_(A*^n^*H_3-*n*_PO_4_)_0.2_ and (CsH_2_PO_4_)_0.8_(HBO_4_)_0.2_.

Compound	Nomenclature
CsH_2_PO_4_	Cs
(CsH_2_PO_4_)_0.8_(BaHPO_4_)_0.2_	Ba
(CsH_2_PO_4_)_0.8_(RbH_2_PO_4_)_0.2_	Rb
(CsH_2_PO_4_)_0.8_(HSO_4_)_0.2_	S
(CsH_2_PO_4_)_0.8_(HMoO_4_)_0.2_	Mo
(CsH_2_PO_4_)_0.8_(HWO_4_)_0.2_	W

**Table 2:** Apparent activation energy values for the studied compounds at low (*T* < 230 °C) and high temperature (*T* > 230 °C).

Compound	*E_act_* (*T* < 230 °C) (eV)	*E_act_* (*T* > 230 °C) (eV)
Cs	1.26	0.65
Mo	0.61	0.19
S	0.42	0.42
W	0.93	0.25
Rb	1.44	0.92
Ba	0.69	0.38
